# Supplementary material for: Are carbon emissions trading and green financial instruments synergistic? -Comprehensive quantitative research based on content analysis
Source: PLoS One. 2024 Mar 7;19(3):e0298601. doi: 10.1371/journal.pone.0298601 (PMC10919601; doi:10.1371/journal.pone.0298601)
Supplement: S1 Data — (ZIP) [file pone.0298601.s001.zip › Data/地级市Z_F工作报告.zip!/║■─╧/╓Ω╓▐╩╨/└┤╫╘╣1⁄2╓┌║┼í╛┬φ┐╦╩2╛▌═°í┐_2003.docx]

各位代表：

　　现在，我代表市人民政府向大会作工作报告，请予审查，并请各位政协委员和其他列席同志提出意见。

　　过去五年工作的回顾

　　过去五年，我们在市委领导下，坚持以邓小平理论为指导，深入实践“三个代表”重要思想，全面履行政府职责，圆满实现了市十一届人民政府确立的经济和社会发展各项预期目标。这五年，是我市经济快速发展，综合实力显著增强的五年；是经济体制和行政管理体制改革取得突破性进展，城乡面貌显著变化的五年；是社会稳定和各项社会事业全面进步，人民生活水平显著提高的五年。

　　——国内生产总值从1997年的226.6亿元增长到2002年的355.3亿元，年均递增10.2%。

　　——财政总收入从1997年的17.72亿元增长到2002年的24.43亿元，同口径年均递增8.06%，其中一般预算收入从1997年的10.97亿元增长到2002年的15.17亿元，同口径年均递增10.03%。

　　——全社会固定资产投资五年累计完成264亿元，年均递增19.5%。

　　——社会消费品零售总额从1997年的87.6亿元增长到2002年的137亿元，年均递增9.4%，五年累计完成567亿元。

　　——进出口总额五年累计完成19.7亿美元，其中出口16.2亿美元。

　　——城镇居民人均可支配收入从1997年的5516元增加到2002年的7920元，年均递增7.5%；农民人均纯收入从1997年的2285元增加到2002年的2934元，年均递增5.1%。

　　——城镇登记失业率控制在4%以内。

　　——人口自然增长率控制在3.7‰以内。

　　一、始终紧扣富民强市的目标，致力于推动经济快速发展

　　推进经济结构调整，构筑产业新框架。五年来，我们大力推进经济结构调整，提高经济运行质量和效益，一、二、三次产业比例从1997年的21.7:45.2:33.1调整到2002年的14.9:48.5:36.6。按照“两跳”思路，大力发展农村特色经济，初步建立了357个初具规模的农产品生产基地，形成了畜禽、竹木、果蔬和粮油四大农产品加工体系，农村“粮猪”型经济结构开始打破。全市共投入资金80亿元，重点支持了一批项目的技术改造，依法对136家国有企业实施破产重组，工业增加值由1997年的89.6亿元增加到2002年的147.4亿元，年均递增11.8%，工业企业净盈利保持年均两位数的增长，2002年实现高新技术产品产值125亿元，年均递增25%。商贸持续繁荣，规划建设了一大批专业市场，芦淞服装市场的改造提升已全面铺开；旅游业发展步伐加快，炎帝陵风景名胜区、大京风景区已被批准为国家4A、3A级旅游区；房地产以及现代物流、信息、交通、邮政、电信、金融、保险、证券服务业都有了大的发展，第三产业增加值从1997年的75亿元增加到130亿元，年均递增10.8%。

　　加强基础设施建设，打造创业新平台。一方面，加快城市基础设施建设。坚持“东提西拓，合拢三角”的总体思路，按照“四高”要求，累计投入建设资金25亿元，大力进行旧城改造和新城拓展。相继改造了十里长街、建设路、新华路；创建了七一路等11条城区样板街；建好了中心广场等6大广场；新建了长江北路等3条高新区主干道；京珠高速公路株洲段、市区“五大通道”等道路建设与改造全面竣工；自来水四厂、霞湾污水处理厂、株洲港千吨级码头二期工程等一批重点工程已经完成；城市快速环道、南郊垃圾处理场、株洲航电枢纽工程等一批重点项目进展顺利；中基洲文化园经过重修，白天象“盆景”，晚上是“夜明珠”；海惠商厦等10多个“烂尾”工程重现生机；湘江生态经济带、“西气入株”前期准备工作正在加紧进行；完成了第六次城市总体规划修编；开展全市“拆违”大行动，已拆除违法建筑47万平方米，工业污染源实现达标排放，城市生态环境有所改善。另一方面，加快农村基础设施建设。共投入2亿多元，对106国道醴陵至茶陵段进行了全线拓宽和提质改造，基本实现乡乡通油路；先后两次荣获全省水利建设竞赛“芙蓉杯”，完成了132座病险水库的除险保安和15个大中型灌区的续建配套；有序进行了农村电网改造，初步实现了“两改一同价”；投入资金2.8亿元，实施项目130多个，重点扶持了13个示范镇的发展，县城扩容提质成效明显，全市城镇化水平达到39.8%。农村基本实现村村通公路、通电、通电视，户户饮上清洁卫生水。

　　深化经济体制改革，建立发展新机制。1、农村改革稳步推进。农村土地延包基本完成；农村税费改革取得成功，农民负担整体减轻。2、企业改革不断深化。着力推进国有企业产权制度改革，市属国有工业企业和国合商业企业改制面分别达到56%、80%；乡镇企业体制创新成效明显，改制面超过95%；国光集团、时代新材股票成功上市；一批大中型企业通过“债转股”共核销不良资产10.8亿元。3、城市管理体制改革取得突破。按照“建管分离，监管分开，块块为主，条条监督”原则和“工作中心转移，管理重心下移”的工作思路，创新了城管体制，城区所有主次干道实现了市场化保洁。城市经营迈出实质性步伐，出让或拍卖城市土地使用权、出租车经营权、户外广告发布权，累计获取建设资金6亿元，城市经营收入正成为“第二财政”。4、财政管理体制改革进展顺利。按照公共财政体制的要求，积极推进了部门预算、零基预算、会计委派、政府采购等各项改革。5、个人住房公积金制度全面建立，住房货币化分配制度基本形成。

　　参与经济全球化竞争，开创开放新局面。一是大力发展外向型经济。坚持“引进来”与“走出去”相结合，利用外资与利用内资相结合，大力发展节会经济，引进了一批国际知名企业和重大国内投资项目，累计合同利用外资3.2亿美元，实际到位外资3.1亿美元。二是进一步扩大对外交往，与海外华人华侨建立了广泛联系，同日本等40多个国家进行了友好交往，先后与乌兹别克斯坦纳沃依、挪威腓特烈斯塔、越南芽庄、南非彼得马里茨堡等5个城市结为友好城市。三是积极争取国家和省里的支持，市领导多次率队拜会国家和省有关部门，争取项目和资金。

　　二、始终坚持以人为本的理念，致力于促进社会全面进步

　　发展各项社会事业，市民素质进一步提高。每届都被评为“全国科技进步先进城市”，五县（市）四区全部进入全国科技工作先进县（市、城区）行列，并促成了“技术创新万里行”在株洲的活动，有4家企业建立了国家级工程中心和企业技术中心，5家企业建立了博士后流动工作站；“跨世纪教育工程”稳步实施，对全市学校布局进行了重大调整，中小学教育资源得到了优化配置，校企剥离工作取得突破，社会化办学初见成效；农村初级卫生保健初步实现，社区卫生服务全面启动，医疗分类管理基本完成，实施了医药收费“阳光工程”，再次跻身“全国卫生先进城市”；计划生育工作连续五年荣获全省先进；“周周乐”等群众性文化活动丰富多彩，反映生活的群众性创作氛围正在形成；参加省九运会“保五争四”的目标顺利实现，申办省十运会取得成功，全民健身活动深入开展，举办中比乒乓球国际对抗赛，产生了良好的反响；广播电视在全省率先实现采编制作播出从模拟向数字化的跨越；连续三年获得全国民族团结进步模范集体称号；修志、老龄、宗教、档案等其他各项社会事业都有新的进步。

　　依法维护社会秩序，稳定大局进一步巩固。深入开展“扫黑除恶”严打整治行动，坚决打击各类犯罪活动，优化了治安环境。认真做好人民群众来信来访工作，有效化解了各类社会矛盾。切实抓好安全生产，全市交通、消防和生产安全形势较为平稳。我们还充分发挥有关行政执法部门的作用，开展了市场经济秩序整顿行动，有力打击了各种扰乱市场经济秩序的行为。

　　健全社会保障体系，群众利益进一步得到维护。全面落实“两个确保”和最低生活保障制度，基本形成了全方位覆盖、一体化管理、社会化发放的保障体系，五年共发放国有企业下岗职工基本生活费6.4亿元，离退休养老金23.6亿元，城市最低生活保障金7228.1万元。积极开展拥军优属活动，两次荣获“全国双拥模范城”光荣称号。大力组织开展救灾救济工作，帮助灾民重建家园、恢复生产；敬老、养老、妇女、儿童福利及残疾人事业都有新的发展。

　　开展文明创建活动，社区面貌进一步改观。提出三年创省文明城市、五年创全国文明城市的奋斗目标，“五创一争”、“讲文明树新风”等活动方兴未艾。学习长沙、常德社区建设经验，把责任落实到基层。有机整合社区资源，城市四区200个居委会合并为108个。健全社区服务网络，安置了1.7万名下岗职工从事社区服务业。石峰区、天元区被评为“全国社区建设示范区”，果园、湘银等7个社区先后成为“全国城市住宅物业管理示范小区”。

　　三、始终按照市场经济的要求，致力于加强政府自身建设

　　自觉接受监督，坚持依法行政，增强了民主法制观念。认真执行人大常委会的决议决定，主动向人大常委会报告工作，自觉接受人大及其常委会的法律监督、人民政协的民主监督以及群众监督和舆论监督。认真办理落实了人大代表建议774件、政协委员提案1397件；开辟《市民与市长》和《101投诉台》等栏目，认真对待和妥善处理群众来信来访，加强了与市民的沟通和联系。与此同时，我们进一步健全行政执法责任制，规范政府及部门的行政行为，行政执法水平有了提高；深入开展“四五”普法，市民法制观念有所增强，法制环境得到改善。

　　切实转变职能，调整工作定位，健全了公共服务体系。按照市场经济的要求，主动实施两个“让位”：把配置资源的主体让位于市场，把生产投入的主体让位于社会。积极推动政府机构改革，工作机构精简到30个，工作人员精减了27.9%。大力改革行政审批制度，行政审批、核准、审核、备案事项由1788项减少到301项，减幅为83.2%，保留的事项中，审批仅21项；大力推进政务公开，政务公开面达到了100%；建立政务服务中心，实行“一站式办公”、“一条龙服务”，提高了办事效率。

　　不断加强学习，改进工作作风，树立了务实廉洁形象。认真组织公务员加强学习，提高了自身素质；认真开展调研督查、“两一·一优”、“首问责任制”等活动，改进了工作作风；充分运用行政监察和审计监督职能，严肃了纪律，促进了廉政建设。

　　回顾五年的工作实践，我们深深体会到，要全面履行好政府工作职责，必须坚持“发展是硬道理”，咬定目标不放松，始终保持一股拼劲；必须坚持改革开放齐头并进，以改革统揽全局，以开放带动发展，始终保持一股闯劲；必须坚持牢牢把握“三个第一”：把思想开放作为株洲的第一开放，把科技创新作为发展的第一推动力，把优化环境作为政府的第一责任，始终保持一股韧劲；必须坚持大力弘扬“开拓奉献，创业争先”的株洲精神，艰苦奋斗，迎难而上，始终保持一股干劲。

　　各位代表，过去五年所取得的这些成绩，是市委正确领导和全市人民共同努力的结果，离不开社会各界的支持、理解和配合。市人大代表、政协委员和各民主党派、工商联、人民团体积极参政议政，踊跃为全市经济和社会发展建言献策；人民子弟兵积极支持和参与地方两个文明建设；社会各界人士和海内外广大朋友，热情关心和帮助株洲发展。谨此，我代表市人民政府向所有关心、支持和帮助株洲发展的同志和朋友们表示衷心的感谢，并致以崇高的敬意！

　　各位代表，我们也清醒地看到，株洲经济和社会发展中还存在许多困难和问题。主要是：经济发展的体制性矛盾仍然比较突出，结构性问题仍然没有得到实质性解决；城乡基础设施仍然比较落后；农民和部分城镇居民收入增幅不大，就业和再就业压力较大，一些群众生活比较困难，社会弱势群体亟需得到关注和扶持；投入渠道不多、总量不大、发展后劲乏力；经济新的增长点不多，财政收支平衡压力较大；社会治安状况有待改善，市场经济秩序需要进一步整顿和规范，等等。究其原因，固然可以找出不少客观因素，从主观上讲，则是由于我们思想观念转变不到位，深化改革的力度不大，扩大开放的程度不深，发展非公有制经济的措施不硬。我们诚恳地接受人大代表、政协委员和社会各界人士对我们的工作提出批评和建议。

　　今后五年工作的指导思想和奋斗目标

　　逆水行舟，不进则退；百舸争流，慢进也是退。聚精会神搞建设，一心一意谋发展，不容许我们有片刻的迟疑，丝毫的懈怠。今后五年政府工作的指导思想是：高举邓小平理论伟大旗帜，全面贯彻“三个代表”重要思想，按照党的十六大精神，以全面建设小康社会统揽工作全局，继续大力实施经济和社会发展“四大战略”，加快推进工业化、城镇化和农业产业化进程，促进经济快速发展，推动社会全面进步。

　　到2007年，我市基本建成全国文明城市、国家园林城市、国家卫生城市、国家环保模范城、中国优秀旅游城市，继续保持全国科技进步先进城市、全国“双拥”模范城等荣誉，进一步巩固我市南方重要交通枢纽地位，为把株洲建成以高新技术产业为先导的新型工业基地和现代物流中心，商贸繁荣、信息通畅、旅游及文化事业发达的区域性中心城市奠定坚实的基础。全市经济和社会发展的主要预期目标是：

　　——国民经济实现跨越式发展。国内生产总值年均增长10.6%；财政总收入稳步增长，一般预算收入年均增长10%。

　　——经济结构调整取得明显成效。一、二、三产业占国内生产总值的比重调整到11：49：40，年均增长速度分别达到3.5%、11.5%、12.5%。

　　——城乡基础设施日臻完善。城镇化水平提高到45%，中心城市功能显著增强，小城镇建设迈出新的步伐。

　　——城乡居民生活质量有较大幅度提高。城镇居民人均可支配收入年均增长8%，农民人均纯收入年均增长5%。

　　——各项事业全面发展，社会保障体系进一步完善。新增劳动力平均受教育年限提高到10年以上，国有企业下岗职工再就业率达到80%，城镇居民登记失业率控制在5%以内。

　　——可持续发展能力不断增强。全社会人口自然增长率控制在6.5‰以内，市区空气质量良好天数每年在280天以上，饮用水质量达标率提高到100%，实现人口增长、资源利用和环境保护与社会发展协调统一。

　　促进经济快速发展和社会全面进步

　　实现今后五年的奋斗目标，我们将始终按照“发展要有新思路、改革要有新突破、开放要有新局面、各项工作要有新举措”的要求，立足政府职能，着力研究和解决事关经济与社会发展全局的重大问题，着力抓好经济发展与社会进步的重大事项。

　　一、着力研究和解决事关经济与社会发展全局的10大问题

　　（一）深化以市场为取向的改革，完善社会主义市场经济体制。主要研究和解决如何加大国有企业改革力度，进一步完善所有制结构；如何健全国有资产管理体制，实现所有者权利、义务相统一；如何建立和完善竞争公平、规范有序的市场体系；如何深化行政管理体制改革，推进事业单位改革，将政府工作的着力点真正放到经济调控、市场监管、社会管理和公共服务上来。

　　（二）加快推进经济结构调整，推动国有经济战略性改组。我市经济体制性、结构性矛盾依然突出，城乡二元结构仍然明显，农村还没有完全打破“粮猪”型经济格局，现代服务业发展滞后。当前，我市产业结构相当于中等收入国家80年代的水平，一、二、三次产业比重短时期内难以形成“三、二、一”的格局，但是，在增长速度上我们要力求做到“三、二、一”。

　　（三）构筑全方位开放格局，开辟出口和招商引资新领域。开放要有新局面，就必须牢固树立内资外资一起要、大项目小项目都需要、出口和进口皆重要的大开放观念，着重研究在新形势下如何提高招商引资的水平。当务之急，我们要加速建设一批工业园区，以园区招商；高质量包装一批项目，以项目招商；为所有投资者提供优质服务，以“商”招商。同时，大力推行招商中介代理制，积极运用跨国购并、境外上市融资等新方式。

　　（四）走新型工业化道路，以信息化带动工业化，以工业化促进信息化。工业化是信息化的物质基础和主要载体，信息化是提升工业化的动力和“引擎”，是加快实现工业化和现代化的必然选择。只有把两者有机结合起来，现代化才能获得快速发展。目前，我市信息化还处于起步阶段，能在多大程度上实现信息化和工业化的结合，将是检验我们在新型工业化道路上是否做到与时俱进的试金石。在加快发展信息产业，引导发展通信、网络、传媒、咨询等信息服务业的同时，我们将进一步完善信息基础设施，为信息化的起飞、工业化的加快推进提供基础和前提。

　　（五）强化多元投入机制，努力扩大投资需求，培育新的经济增长点。扩大社会投资需求，必须研究如何全面强化多元投入机制。我们在积极争取国家资金尤其是国债资金支持、吸引外资的同时，坚定不移、毫不动摇地鼓励、支持和引导非公有制经济发展，废除一切有碍于非公有经济发展的政策规定，给予非公有经济与国有经济一视同仁的政策，引导民营企业参与国有企业的改革、改组和改造，支持民营企业进入基础设施建设、社会事业等领域，努力形成企业以民营为主、资金以民间为主、事业以民办为主的新格局。

　　（六）完善人才支撑体系和技术创新体系，切实提高自主创新能力。人才是第一资本，科技是第一生产力，现代经济的竞争从某种意义上说就是人才和科技的竞争。如何完善人才的社会评价机制、人才激励机制以及技术创新和服务体系，不拘一格选人才、用人才，真正做到唯才是举、唯才是用，从而让所需要的高科技人才和各类专门人才进得来、留得住、用得上，构筑起株洲人才新高地，不断增强株洲的技术创新能力，创造一批新的株洲“第一”和诞生具有较强市场竞争力的株洲品牌，是我们面临的又一重大课题。

　　（七）加大经营城市力度，打造城市特色品牌，提高城市综合竞争力。城市是最大的国有资产，经营国有资产的重中之重就是经营城市。必须注重研究激活城市资产的商品属性，在努力做强产业、做优服务的同时，着重做好经营城市土地这篇大文章，力争经营收入每年有较大幅度增长，推动城市基础设施不断完善，进而实现产业、基础设施、环境“三箭齐发”，构筑株洲较强的综合竞争力。

　　（八）加强社区建设，促进城市人居环境建设和精神文明建设。区是城市最基本的单位。随着城市居民由“单位人”向“社会人”转变，社区建设日益显得重要。务必在搞好典型引路，加大财政投入的同时，进一步调动和发挥街道办事处、居委会以及社区所在地企业、单位和居民的积极性，形成建设社区的强大合力，重点在完善社区功能、改造美化社区、丰富居民社区文化生活等方面加大工作力度。

　　（九）理顺市和区以及县（市）和乡（镇）管理体制，调动和发挥多层面的积极性。在新形势下，市和区以及县（市）和乡（镇）之间的行政管理体制出现一些不适应的方面，主要表现为职责不十分明确，权利和责任不完全一致。比方说，市政府与区政府之间的城市建设和管理权限，县（市）与乡（镇）的行政管理体制，等等，还有很多文章要做。

　　（十）扩大就业，促进再就业，努力提高低收入者生活水平。就业是民生之本，就业已经成为一个十分突出的社会问题，而且在相当长的一个时期内，就业和再就业的压力会越来越大。就业问题不解决，将引发诸多社会问题。政府将在引导转变就业观念、健全就业机制、广开就业门路等方面作出不懈的努力，最关键的就是依靠发展，增加就业机会，从根本上解决问题。

　　二、着力抓好经济发展和社会进步的10大事项

　　（一）以农村基础设施建设为主线，加快农业、农村现代化进程。重点抓好城市防洪工程，湘江、渌江、米水、洮水四大水系整治，病险水库除险保安，大型优质稻基地建设，骨干渠道整治等，从整体上提高防汛抗灾能力；完成106国道株洲段全线提级改造，积极做好前期准备工作，力争衡炎高速、上瑞高速株洲段尽早开工，启动县到乡支线新改建工程，力争五年内100%的乡镇通油（砼）路，50%的行政村通油（砼）路，全市公路技术等级率达到80%；支持茶陵建设洮水水库，完成农网二期改造，实现城乡“同网同价”；切实加强小城镇建设，加快农村城镇化进程；进一步抓好农业综合开发，力争把开发项目建设成为农业现代化示范工程。

　　（二）以完善城市功能为内核，加快“双百”城市创建步伐。市政设施方面：突出“一环、一带、两厂、四路”建设。一环即城市快速环道，五年内北环线全面竣工，东环线全线拉通，建宁大桥建成通车；一带指湘江生态经济带，在河西完成石峰大桥至株洲县城段的建设；两厂指建好龙泉污水处理厂及白石港污水处理厂；四路指对天易公路美化亮化，建成120米宽的株洲大道，拉通庐山路、嵩山路、珠江南路，改造完毕建设路、新华路，支持城市四区按每年一路的标准搞好样板街建设。园林绿化方面：着力抓好天台、流芳园等公园和滨江、红港路、泰山、天易等广场建设，至2007年，力争城市绿地率在35%以上，城市绿化覆盖率提高到40%以上，人均公共绿地面积提高到8平方米以上。公用事业方面：建成河西220KV和向阳村110KV变电站，抓好城区电网改造、通讯网络完善和“西气入株”工程等，增强水、电、气、通讯网络的供给和保障能力。

　　（三）以国道和省道为骨架，构建株洲大交通体系。力争京广高速铁路在株洲布线设站；按照“建设主骨架，改善干支网，提高等级率，扩大覆盖面”的思路，重点打造“三纵十二横”，实现株洲三小时，县（市）一小时的公路快速交通圈；以株洲航电枢纽工程建设为契机，加快湘江千吨级航道三期开发，与沿江邻近地市一道建成直达出海口的“快速航运”体系；完成河西天元汽车站、醴陵立三汽车站、攸县汽车中心站建设，进一步完善城乡站场设施。

　　（四）以“一区三园”为平台，大力发展高新技术产业。按照“10年再造一个株洲”的总体构想，依托“一区三园两基地”发展平台，继续把高新区作为全市重点区域，促进全市高新技术产业快速发展。紧紧抓住我市被列入全国制造业信息化重点城市的机遇，以信息化带动工业化，加快发展科技含量高、经济效益好的生物制药、新材料、健康食品等新兴产业。注重运用高新技术和先进适用技术改造和提升传统产业，重点扶持和发展有色冶金、交通设备及配件等产业，培育壮大劳动密集型产业。支持醴陵建设陶瓷工业园，做大做强陶瓷产业，形成门类齐全、结构合理、专业配套的现代化陶瓷产业体系，把陶瓷产业建设成为全省的标志性产业。

　　（五）以市场建设为依托，推进服务业现代化进程。抓紧建好长株潭现代物流中心，加快株洲汽车超市、汇亚国际服装博览中心等大型项目建设，按照“两改一提升”的方针，加快建设芦淞服饰物流中心；促进连锁经营、电子商务、物流配送以及金融、证券、房地产、信息等现代服务业快速发展；重点建好炎帝陵、桃源洞、云阳国家森林公园、空洲岛旅游休闲度假区、酒埠江风景区、大京风景区、仙庾风景区，开发工业旅游，打造以炎帝文化为代表的株洲旅游新形象。

　　（六）以载体建设为支撑，全面发展各项社会事业。高质量完成“普九”，基本普及高中阶段教育，进一步办好各级各类教育，支持中南林学院、株洲工学院、株洲师专和几所高等职业技术学院办大办强，支持醴陵市筹建湖南陶瓷学院。以承办省十运会为契机，建好体育中心，完善体育设施，当好东道主，办好十运会。继续发展卫生服务机构，新建河西医院、卫生医药科教中心、疾病监测中心和市中医院门诊大楼，完善对重点传染病、慢性病的预防和控制体系，提高全民健康水平；全面实施卫生区域规划，形成小病在社区，大病进医院的卫生服务格局。积极创造条件，启动新世纪乐园、文化艺术中心、科技馆等设施建设，为各类事业的发展提供基础和支撑。

　　（七）以农产品加工业为突破口，推动“三化”进程。继续大力实施“种苗工程”，依托资源和传统优势，以体制创新和机制创新为动力，重点发展竹木业、肉食、油脂、果蔬、米业等五大优势产业，扶持一批龙头加工企业做大做强，规划和建好一批农产品加工工业园，大力发展农村工业和服务业，加快农业产业化、农村工业化、农村城镇化进程。

　　（八）以加强公共管理为重点，维护正常的社会秩序。加强市场监管，规范市场经济秩序，特别是加大对各种涉税违法犯罪行为的查处力度，教育和引导纳税人合法经营，照章纳税；强化社会治安综合治理，营造安定祥和的治安环境；进一步做好安全生产工作，尽最大可能防止和减少重特大事故发生；加强城市管理，尽快推行城管综合执法，加大市容市貌整治力度，为全市人民创造整洁文明的生产和生活环境。

　　（九）以促进人与自然和谐为目标，继续大力实施可持续发展战略。认真贯彻《人口与计划生育法》，提高人口素质，保持低生育水平；认真贯彻2001—2010年全市妇女发展规划和儿童发展规划，促进妇女儿童事业持续发展。切实保护耕地，确保耕地占补平衡；抓好矿产资源开采管理，坚决遏制破坏生态的行为；切实加强植树造林、退耕还林、荒山治理和水土保持工作，抓好60万亩退耕还林、400万亩生态公益林建设，把森林覆盖率提高到56%。以清水塘地区为重点，加大城市大气污染和水环境综合整治力度，进一步改善市区空气和饮用水质量。

　　（十）以规范市场经济道德秩序为着力点，完善社会信用体系。人无信不立，企业无信不盛，国家无信不强。在日趋激烈的市场竞争中，诚信是安身立命之本、成长发展之基、竞争取胜之宝。建立诚信，我们将在重点抓好市场经济秩序，特别是金融秩序和房地产市场秩序整顿的同时，大力开展诚信教育，倡导信用观念，建立健全社会信用体系，完善信用评级制度，建立信用信息披露制度，规范发展信用中介，形成合理的失信约束惩戒制度。

　　努力实现全面建设小康社会的良好开局

　　2003年是市十二届人民政府任期的第一年，也是贯彻党的十六大精神，全面建设小康社会，加快推进社会主义现代化的第一年。做好今年的各项工作，对于保持新世纪新创业的良好来势，为加快实现第三步战略目标开好头、起好步，具有十分重要的意义。按照今后五年工作的总体要求，今年我市经济和社会发展的主要预期目标是：国内生产总值达到390亿元，比上年增长11%，其中一、二、三产业增加值分别增长3.5%、12%、12.5%；财政总收入稳步增长，其中一般预算收入确保增长10%；城市居民人均可支配收入增长8%，农民人均纯收入增长5%；城镇登记失业率控制在5%以内；各项社会事业都有新的发展。

　　围绕上述目标，我们将在全面做好各项工作的同时，突出以下几个重点：

　　抓改革。坚持用改革的办法解决发展中的问题，以改革的新突破推动经济和社会的大发展。年内重点抓好以下几项改革：一是农村各项改革。以《农村土地承包法》和中共中央《关于土地流转问题的若干意见》为指导，进一步规范农村土地使用权流转，完善统分结合的双层经营体制；以农村乡镇机构改革和农村中小学布局调整为重点，进一步巩固深化农村税费改革及其各项配套改革成果；以村帐乡代管为突破口，进一步推进村务公开，努力扩大基层民主，逐步化解乡村债务。二是国有企业产权制度改革。务必全面深化，重点突破，坚定不移搞改革，竭尽全力搞改制；对国有资本退出领域，则降低准入门槛，实行一视同仁，鼓励和支持个体、私营、民营经济成份进入。同时，引导规模企业建立现代企业制度，按公司法要求规范运作。三是行政管理体制改革。主要有四项：1、行政审批制度改革。重点是巩固前两年改革成果，进一步办好政务服务中心。2、财政管理体制改革。按照建立公共财政的要求，以综合财政预算为支撑，全面推进部门预算、零基预算、会计集中核算和政府采购制度，扩大会计集中核算范围，加快向国库集中支付核算过渡步伐。3、城市管理体制改革。按照责权利相统一，人（财）随事转的原则，还权于基层，放权于基层，进一步完善城市管理体制。4、事业单位管理体制改革。按照脱钩、分类、放权、搞活的原则，大力精简机构和人员，全面创新管理体制。

　　抓发展。发展是硬道理，是解决前进中的困难和问题的根本所在。今年，我们将切实抓好以下这些重点工程和项目，以此带动全市经济和社会的快速发展。城市基础设施方面：继续推进城市快速环道二期工程，抓好建宁大桥、东环线和东湖立交的建设；完善高新区栗雨和天台工业园的基础设施；支持城市四区建好铁西路等四条样板街；加快株洲航电枢纽、南郊垃圾处理场的建设步伐；做好前期工作，迎接“西气入株”；改造一、二、三水厂，完善四水厂配套管网；启动龙泉污水处理厂和湘江生态经济带景观防洪道路建设；完成工业污染重点治理项目10个；拆除各类违章建筑50万平方米，加快公共绿地建设，提升城市园林绿化品位，力争成功创建省级园林城市。产业项目方面：推动一批重点工业项目早日竣工投产；支持汇亚国际服装博览中心、芦淞服饰物流中心、汽车超市、禽畜水产品大市场等一批三产业重点项目的建设；启动市体育中心等项目建设，继续抓好市疾病监测大楼的建设。农村基础设施方面：力争衡炎高速和上瑞高速株洲段开工建设，年内完成106国道醴陵北段和攸县菜花坪至茶陵县段的建设，启动攸县米水大桥及绕城线改造工程，确保网酒公路、峦漕公路沥青（砼）路面配套工程、万大线水泥（砼）路面配套工程、株洲县渌江大桥及连接线全面竣工，抓紧进行株洲县龙潭乡至攸县贾山乡三级公路、炎陵县沔渡至桃源洞三级公路改造，启动总里程为328公里的县乡公路提升改造；以株洲县城段为重点，切实抓好湘江防洪工程建设；进行农网二期改造，启动县城电网改造；重点抓好工业园区建设，继续推进退耕还林和优质稻基地建设，增强农业和农村经济发展后劲。

　　抓稳定。稳定是改革与发展的前提和基础。进一步采取有力措施，改善治安环境，保持社会稳定，人民政府义不容辞，责无旁贷。重中之重，就是要始终保持对犯罪分子的高压态势，重点打击严重威胁人民群众生命财产安全的暴力犯罪、有组织犯罪、黑恶势力犯罪和重大经济犯罪以及入室盗窃、抢劫、抢夺等多发性侵财犯罪；深入开展“四五”普法活动，提高全市人民的法制意识；切实做好人民来信来访接待工作，坚决防止可能造成严重影响的重大群体性事件发生；认真做好就业和再就业工作，年内新建成23个劳动保障服务站和130个劳动保障服务点，改造或新建11个社区服务中心、10个再就业市场和覆盖全市的劳动力市场网络平台，力争再就业率提高到65%以上，城镇登记失业率控制在5%以内；全面落实“两个确保”措施，确保国有企业下岗职工基本生活费和离退休人员养老金及时足额社会化发放；高度关注残疾人、丧失劳动能力的人和农村“五保户”、城乡特困家庭等社会弱势群体，帮助他们解决生产生活中的实际困难；认真做好生产安全、交通安全、消防安全工作，防止发生重特大安全事故。

　　抓环境。优化环境是政府的第一责任。今年我们将继续采取有力措施，把优化环境工作推向一个新台阶。牢固树立“无外不快、无外不活”的思想，“亲商、招商、安商、富商”，营造包容大气的开放环境；充分发挥工商、税务、物价、建工、文化、卫生、质监、海关、药品监督、检验检疫等部门的职能作用，进一步整顿和规范市场经济秩序，营造文明有序的经营环境；整顿社会信用秩序，积极创建金融安全区，进一步完善企业信贷担保体系，营造诚实守信的金融环境；充分尊重人民群众的首创精神，不断拓宽与人民群众的沟通渠道，营造团结向上的舆论环境；改进工作作风，提高办事效率，营造高效便民的服务环境。

　　各位代表，我们将一如既往自觉坚持党的领导，全面贯彻实施市委的决策部署；一如既往坚决执行人民代表大会及其常委会的决议决定，认真听取并尊重人民政协、各民主党派、工商联以及其他社会各界人士的意见，加强同法院、检察院、军队、武警等国家机关以及工会、共青团、妇联等群众团体的密切联系；一如既往团结和依靠全市人民，充分发挥人民群众的主动性、积极性、创造性；一如既往真诚面对并接受来自各个方面的监督，保障人民群众依法管理国家和事务的权利。我们还知道，全面做好政府工作，迫切需要进一步解放思想，更新观念，注重依靠经济和法律手段管理公共事务，运用市场手段推动经济和社会发展；迫切需要大力推进依法行政，坚决纠正一些执法人员以罚代管、以情代法、徇私枉法等不法行为，做到公正廉洁、依法办事、文明执法；迫切需要不断提高自身思想政治素质和业务水平，大力推行依法行政，提高工作水平。

　　各位代表、同志们，我们有幸处于一个大改革、大开放、大发展的时代，历史的责任、人民的重托赋予了我们庄严而神圣的使命。让我们在市委的领导下，高举邓小平理论伟大旗帜，全面贯彻“三个代表”重要思想，与时俱进，开拓创新，加快全面建设小康社会的步伐，在株洲经济和社会发展史上谱写出新的篇章！
